# Development of SPACE-II for rapid sample exchange at SPring-8 macromolecular crystallography beamlines

**DOI:** 10.1107/S2059798320000030

**Published:** 2020-01-31

**Authors:** Hironori Murakami, Kazuya Hasegawa, Go Ueno, Naoto Yagi, Masaki Yamamoto, Takashi Kumasaka

**Affiliations:** a Protein Crystal Analysis Division, Japan Synchrotron Radiation Research Institute, 1-1-1 Kouto, Sayo-cho, Sayo-gun, Hyogo 679-5198, Japan; b Advanced Photon Technology Division, RIKEN SPring-8 Center, 1-1-1 Kouto, Sayo-cho, Sayo-gun, Hyogo 679-5148, Japan

**Keywords:** macromolecular crystallography, synchrotron beamlines, sample changer, SPACE-II, SPring-8

## Abstract

Reducing the sample-exchange time is a crucial issue in maximizing the throughput of macromolecular crystallography (MX) beamlines because the diffraction data collection itself is completed within a minute in the era of pixel-array detectors. To this end, an upgraded sample changer, SPACE-II, has been developed on the basis of the previous model, SPACE (SPring-8 Precise Automatic Cryo-sample Exchanger), at the BL41XU beamline at SPring-8. SPACE-II achieves one sample-exchange step within 16 s, of which its action accounts for only 11 s, because of three features: (i) the implementation of twin arms that enable samples to be exchanged in one cycle of mount-arm action, (ii) the implementation of long-stroke mount arms that allow samples to be exchanged without withdrawal of the detector and (iii) the use of a fast-moving translation and rotation stage for the mount arms. By pre-holding the next sample prior to the sample-exchange sequence, the time was further decreased to 11 s in the case of automatic data collection, of which the action of SPACE-II accounted for 8 s. Moreover, the sample capacity was expanded from four to eight Uni-Pucks. The performance of SPACE-II has been demonstrated in over two years of operation at BL41XU; the average number of samples mounted on the diffractometer in one day was increased from 132 to 185, with an error rate of 0.089%, which counted incidents in which users could not continue with an experiment without recovery work by entering the experimental hutch. On the basis of these results, SPACE-II has been installed at three other MX beamlines at SPring-8 as of July 2019. The fast and highly reliable SPACE-II is now one of the most important pieces of infrastructure for the MX beamlines at SPring-8, providing users with the opportunity to fully make use of limited beamtime with brilliant X-rays.

## Abstract 2

A rapid and reliable sample changer, SPACE-II, has been developed at the SPring-8 macromolecular crystallography beamline BL41XU. It enables samples to be exchanged in 16 s, of which its action accounts for only 11 s. Two years of operating SPACE-II demonstrated that the average number of sample exchanges per day was increased by 40% compared with the previous model, and it had an error rate of only 0.089%.

## 1. Introduction   

The automation of data collection at macromolecular crystallography (MX) beamlines is important for the efficient use of the limited beamtime. One key apparatus for automation is a sample-changer robot; this enables samples to be exchanged without entering the experimental hutch and such robots are installed at most MX beamlines nowadays. The use of sample changers not only reduces the overhead time owing to sample exchange but also reduces the labor required from the users, therefore allowing data collection from the vast number of crystals that are obtained in large-scale screening for difficult targets as well as high-throughput ligand screening for drug discovery (Wasserman *et al.*, 2012 ▸; Tsai *et al.*, 2013 ▸). Another advantage is the reduction in human error which could damage the beamline apparatus, such as the beam stop and collimator. Sample changers also enable data to be collected by remote access, which allows a synchrotron beamline to be used from a user’s home laboratory, reducing the time that the user spends on traveling and reducing travel expenses (Soltis *et al.*, 2008 ▸; Ueno *et al.*, 2016 ▸). In the ultimate case, sample changers enable fully automated data collection (Ueno *et al.*, 2004 ▸; Hiraki *et al.*, 2010 ▸; Wasserman *et al.*, 2012 ▸; Svensson *et al.*, 2015 ▸; Hirata *et al.*, 2019 ▸).

A number of sample changers have been developed since the early 2000s at MX beamlines worldwide. They are categorized into two main streams, as reviewed by Ferrer *et al.* (2013 ▸). One stream involves the use of general-purpose multi-axis robots, such as the Stanford Automated Mounter (SAM) robot (Cohen *et al.*, 2002 ▸; Russi *et al.*, 2016 ▸), the Automated Crystal Transport Orientation and Retrieval (ACTOR) robot (Muchmore *et al.*, 2000 ▸), the Cryogenic Automated Transfer System (CATS; Jacquamet, Ohana, Joly, Legrand *et al.*, 2004 ▸; Jacquamet *et al.*, 2009 ▸), BART (McAuley *et al.*, 2015 ▸), Marvin (Cianci *et al.*, 2017 ▸) and FlexED8 (Papp, Felisaz *et al.*, 2017 ▸). The flexible arms of these sample changers have a wide working area and can pick up sample pins from a large liquid-nitrogen Dewar that can accommodate a large number of sample cassettes. The other stream is sample changers that are specialized for the exchange of cooled protein crystals, such as SC3 at the ESRF (Cipriani *et al.*, 2006 ▸), an ALS-style robot (Snell *et al.*, 2004 ▸; Cork *et al.*, 2006 ▸) and DORIS (Karain *et al.*, 2002 ▸). These sample changers are composed of a mount arm and an automated Dewar that can convey sample pins to a designated position. In either case, a bottleneck to rapid sample exchange is the requirement for two cycles of motion, *i.e.* dismounting a sample on a goniometer and mounting a new sample in the next cycle, which increases the time taken to exchange samples. To fix this problem, a double tong was developed for a Photon Factory (PF) automated mounting system (PAM; Hiraki *et al.*, 2008 ▸). It reduced the time taken for sample exchange to 10 s because the next sample is already being gripped prior to the start of sample exchange. The CATS sample changer from IRELEC also implemented double tongs to reduce the time for sample exchange (https://www.irelec-alcen.com). FlexED8, which was recently developed at the EMBL Grenoble for the former EMBL–ESRF–India beamline BM14 at the ESRF, achieves a nominal sample-exchange time of 5 s by implementing a double gripper, where the time taken to move the mount arm between the sample storage and the gonio­meter might not be included (Papp, Felisaz *et al.*, 2017 ▸). Another approach to exchange samples in one cycle is to use one of the robot axes as a spindle axis of the goniometer, which was attempted by G-Rob on beamline FIP-BM30A of the ESRF (Jacquamet, Ohana, Joly, Borel *et al.*, 2004 ▸). The drawback of this approach was the large sphere of confusion (SOC) of the robot axis compared with that of a designated goniometer. Therefore, dynamic correction was needed to reduce the SOC during data collection, which is difficult in the case of shutterless data collection performed using a high-frame-rate detector. However, this problem can be fixed by attaching a high-accuracy air-bearing goniometer at the tip of a multi-axis robot, which was demonstrated by the development of RoboDiff on the ESRF beamline MASSIF-1 (Nurizzo *et al.*, 2016 ▸).

At SPring-8, a sample changer, SPring-8 Precise Automatic Cryo-sample Exchanger (SPACE), has been developed (Ueno *et al.*, 2004 ▸). The unique feature of SPACE is that it utilizes a special sample pin possessing left-hand and right-hand screw threads on either side of it, and sample pins are transferred between the mount arm and goniometer by a simple rotation of the mount arm. The use of this screw pin enables sample pins to be mounted on a goniometer in a highly reproducible manner, and it allows a type of automatic data collection called ‘two-step operation’, which is composed of daytime crystal evaluation and nighttime data collection. In the first step, all crystals are evaluated by collecting several diffraction images after the crystals have been manually centered by a beamline operator. The center coordinate of each sample is stored in the beamline database D-Cha (Okazaki *et al.*, 2008 ▸) and is used for the automatic centering in the second step, in which fully automatic data collection is conducted by using selected crystals on the basis of the evaluation in the first step. Although two-step operation is useful for high-throughput data collection and contributed to structural genomics research in the Protein 3000 project (Yokoyama *et al.*, 2000 ▸), requests to use general sample pins, such as SPINE-style, ALS-style and SSRL/SAM-style pins, had increased, especially from users working on challenging targets. Moreover, compatibility between sample pins and sample cassettes among MX beamlines would be of benefit to the user community. Therefore, a novel sample holder that can grip conventional sample pins was developed (Murakami *et al.*, 2012 ▸). Since the new sample holders can be replaced with the original sample holders for the screw pins in 10 min, both the general sample pins and the screw pins can be used on one beamline. This adaptation of SPACE to general magnet pins has increased the use of SPACE to 100% at BL32XU (Hirata *et al.*, 2013 ▸) and BL41XU (Hasegawa *et al.*, 2013 ▸), where data collection for challenging targets such as membrane proteins is conducted using a high-flux microbeam.

However, there were still issues to cope with; one such issue was the time taken for sample exchange. The installation of a pixel-array detector (PAD) can decrease the typical data-collection time to the order of minutes. However, the time for sample exchange had been about 1 min, which is equivalent to the total exposure time. Therefore, speeding up sample exchange was necessary to make full use of high-speed data collection by a PAD. Another issue was the sample capacity. Only four Uni-Pucks, with a total of 64 sample pins, could be stored in SPACE, which meant that the Uni-Pucks needed to be exchanged in one beamtime. To fix these problems, we developed SPACE-II on the basis of the conventional model, and achieved sample-exchange times of 16 s, of which the action of SPACE-II accounts for 11 s, as well as doubling the sample capacity. Since the installation of SPACE-II at BL41XU in April 2017, it has contributed to the efficient use of beamtime, with an error rate of 0.089%.

## 2. Development of SPACE-II   

### 2.1. Overall architecture   

**Figure 1:**
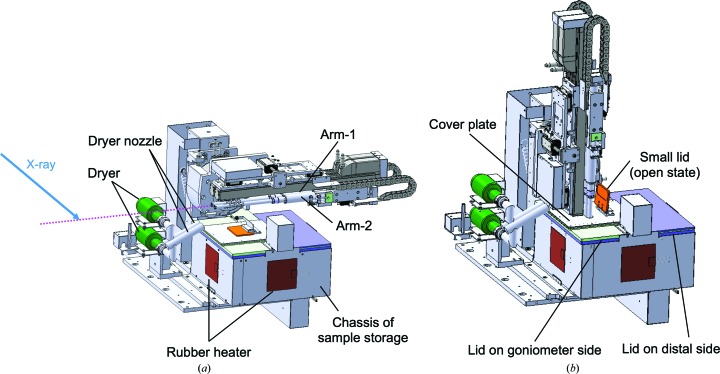
3D drawing of SPACE-II. The mount arms are oriented to the goniometer in (*a*). They are oriented to the sample storage and extended into the liquid-nitrogen Dewar in (*b*). The magenta dotted line in (*a*) indicates the direction of the goniometer spindle axis.

**Figure 2:**
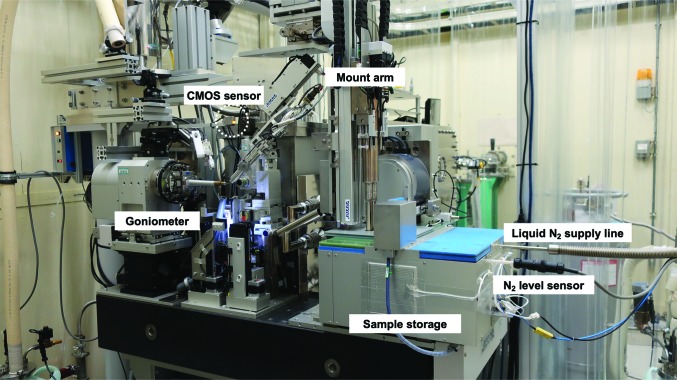
SPACE-II installed on BL41XU at SPring-8. It is equipped on a granite diffractometer table. The safety light curtain described in Section 2.5 is removed to clearly show the overall shape of SPACE-II.

Fig. 1 ▸ shows the overall architecture of SPACE-II, which is similar to the previous model, SPACE; it is composed of twin arms and sample storage. Although the mount arms have a long stroke and the sample storage is larger in SPACE-II, as described in the following section, it is still compact enough to be located on a diffractometer table (Fig. 2 ▸). A translation axis for the removal of SPACE-II in the upstream direction was also implemented (E-stage; Evacuation stage), but it is not used in usual operation because SPACE-II never interferes with a detector or other devices on the diffracto­meter. It is mainly used so that the beamline staff can easily access the apparatus near the sample for maintenance purposes.

### 2.2. Mount arm   

**Figure 3:**
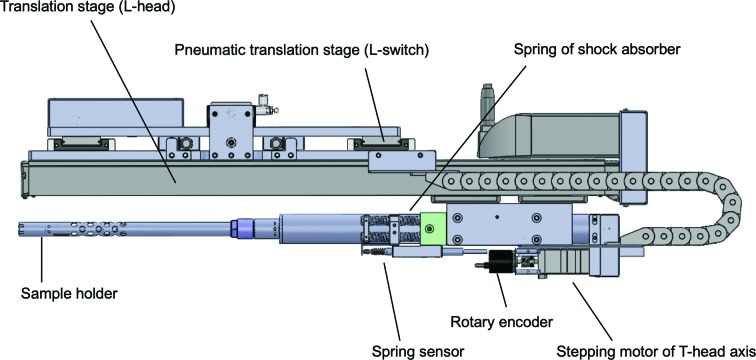
3D drawing of a mount arm viewed from the side. The arms are oriented to the goniometer direction.

The most characteristic feature of SPACE-II is the implementation of twin arms; one arm, Arm-1, is used for mounting a new crystal, and the other arm, Arm-2, is used for dismounting a crystal from the goniometer. These two arms are located on the same rotation stage (T-arm; Theta arm). The stage orients the twin arms from the sample storage to the goniometer and vice versa by rotating 90°. The positions of these two arms can be switched by 48 mm along the incident X-ray beam direction by using a pneumatic translation stage (L-switch; L-head switch) (Fig. 3 ▸), *i.e.* when one arm is just in front of the goniometer the other arm is offset by 48 mm. Each mount arm has a translational axis (L-head; Linear-head) to independently access the goniometer or sample cassettes. The stroke of the L-head is 550 mm, which allows us to locate SPACE-II at a maximum of 550 mm from the sample position, which is distant enough so as not to interfere with the detector (PILATUS3 6M, replaced by an EIGER 16M in April 2018), even at the minimum detector distance of 150 mm. On the translation table of the L-head axis, a sample holder is equipped via a sliding stage whose resting position is fixed by springs (Fig. 3 ▸) so that it acts as a shock absorber. The deviation from the resting position is monitored by a linear gauge (LGB-110; Mitutoyo, Japan; Fig. 3 ▸), because an extension or contraction that is too large indicates that a problem has occurred in picking up or releasing a sample pin. In the case of such an extension or contraction, SPACE-II is forced to stop immediately.

**Figure 4:**
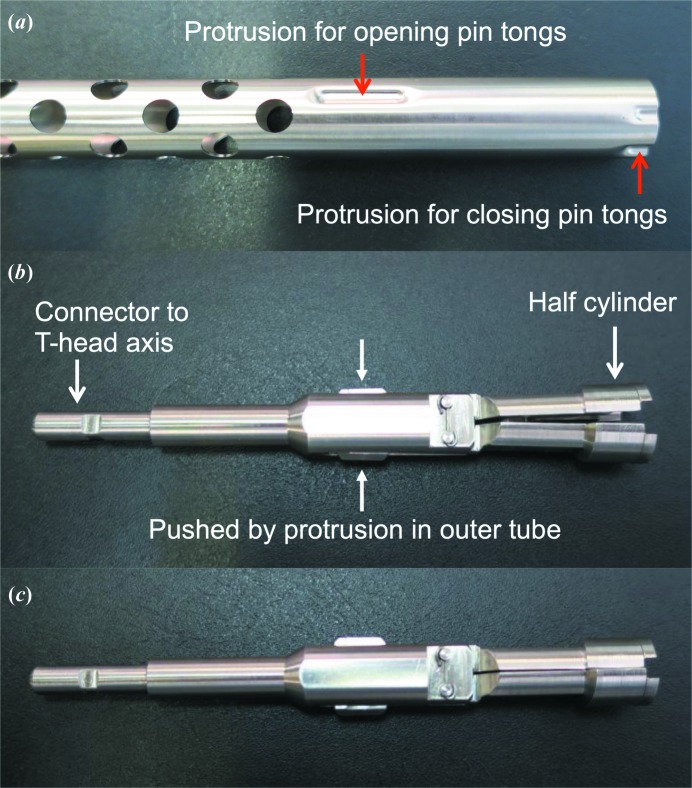
Photographs of the new outer tube (*a*) and pin tongs (*b*, *c*). (*b*) and (*c*) show open and closed states, respectively.

The design of the sample holder is the same as that of SPACE (Murakami *et al.*, 2012 ▸), *i.e.* it consists of an outer cylindrical tube and cylindrical pin tongs installed in the outer tube. The outer tube is made of stainless steel and is fixed on the main part of each of the twin arms. The pin tongs are composed of two half-cylindrical parts facing each other, and their opposite sides are connected to a rotation axis (T-head; Theta head) (Fig. 4 ▸). The stepping motor of the T-head axis is located at the distal side of the mount arm, as shown in Fig. 3 ▸. Since the outer radius of the half cylinders of the pin tongs changes gradually along their circumference and the outer tube has protrusions, a simple rotation of the tongs enables a hinge-like motion for gripping or releasing sample pins. The basic design of the pin tongs is the same as that of the previous model, but it was modified to improve the integrity of sample release in SPACE-II. In the previous model, the half cylinders were connected via a plate spring that produces force to open the pin tongs (Murakami *et al.*, 2012 ▸), but a problem sometimes occurred in which a sample pin that had become tightly attached owing to ice formation between the tong and sample pin could not be released. Since this problem indicated that the plate spring did not produce sufficient force to release the pins, the outer tube and the pin tongs in the latest model were modified so as to be opened by the principle of a lever; that is, there are other protrusions on the outer tube at 30 mm from the tip of the tube, and they force the pin tongs to open by pushing the lever at the middle of the pin tong (Fig. 4 ▸). Since the rotation angle of the pin tongs is directly related to the opening of the pin tongs, it is monitored by a rotary encoder (C6A2-C; OMRON, Japan) that is attached to the stepping motor of the T-head axis (Fig. 3 ▸). Too much rotation means that no sample pin is being gripped, and too little rotation means that opening or closing of the pin tongs failed, which is mostly caused by the tight attachment of a sample pin to the pin tongs via ice.

Another upgrade from the conventional SPACE is the use of a direct-drive motor for the T-arm and a robo-cylinder for the L-head that increase the speed of the mount arms. The direct-drive motor DDA-LH18C (IAI, Japan) was used for the T-arm and the robo-cylinder RCP6 (IAI, Japan) was used for the L-head.

By the implementation of the long stroke arms, the only possible interference between SPACE-II and other devices is the nozzle part of the cryocooler, which is kept 6 mm from the sample position during data collection. Since the diameter of the outer tube of the sample holder is 15 mm, removal of the nozzle is needed prior to sample exchange.

### 2.3. Sample storage   

**Figure 5:**
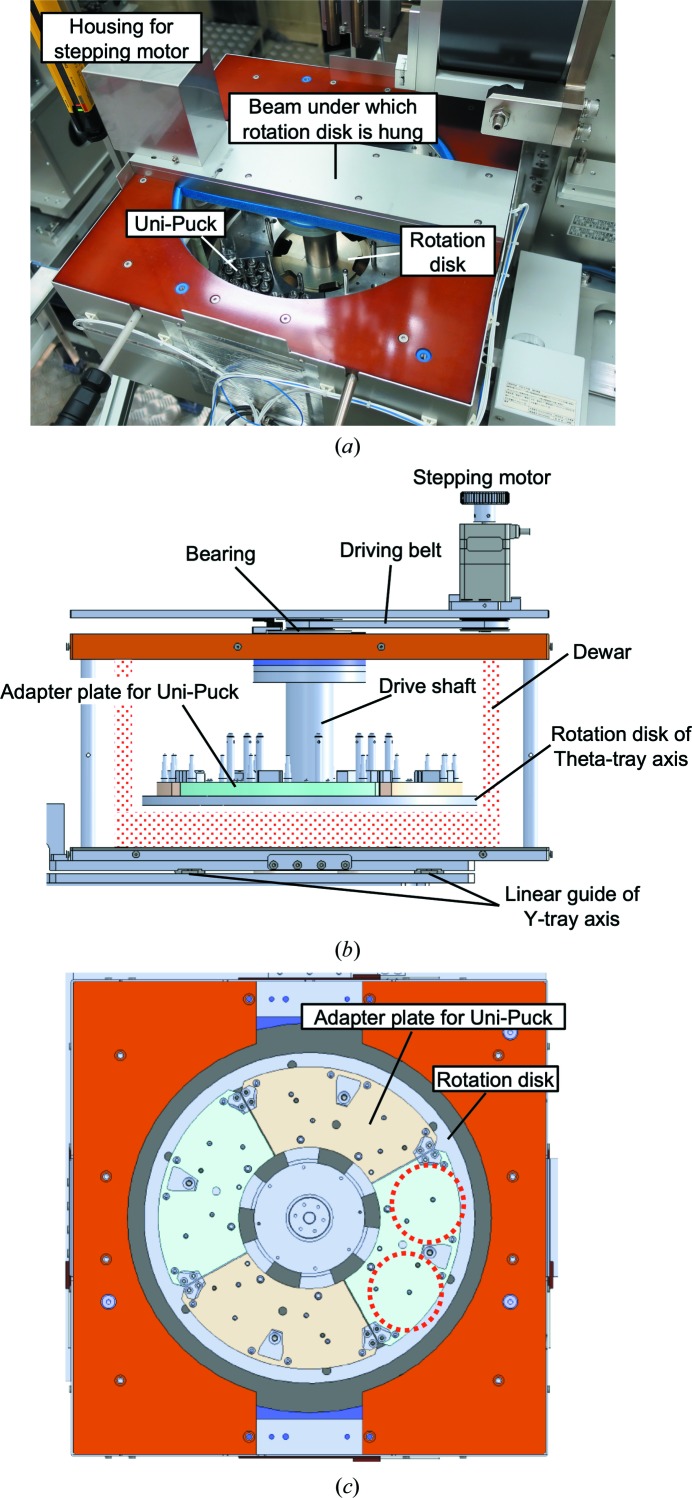
(*a*) Photograph of the sample storage. Two Uni-Puck cassettes are installed. The lids are removed and liquid nitrogen is not filled for clarity. (*b*) A 3D drawing showing a cross section of the sample storage. The liquid-nitrogen Dewar is depicted as a red mesh pattern. (*c*) A 3D drawing showing the top view of the sample storage. The two red dotted circles indicate the positions of Uni-Pucks mounted on one of the adapter plates.

The sample-storage part of SPACE-II is a square chassis of 400 × 400 × 210 mm in which a cylindrical liquid-nitrogen Dewar is installed (Figs. 5 ▸ *a* and 5 ▸ *b*). To increase the number of cassettes accommodated in SPACE-II within the limited available space, a combination of a rotation disk (Theta-tray) and a translation axis (Y-tray) was adopted to convey samples to the position of the mount arm (Fig. 5 ▸ *b*). The rotation disk was installed in the liquid-nitrogen Dewar because there was not enough space to equip it under the Dewar, owing to the narrow distance of 400 mm between the spindle axis and the diffractometer table on the SPring-8 MX beamlines. A maximum of eight Uni-Pucks can be loaded on the rotation disk via four adapter plates that can fix two Uni-Pucks on each (Figs. 5 ▸ *b* and 5 ▸ *c*). The rotation disk is hung from the central beam of the storage chassis (Figs. 5 ▸ *a* and 5 ▸ *b*). To avoid interference with the mount arms, the driving motor of the rotation disk is located at the edge of the chassis, and the disk is rotated via a driving belt (Fig. 5 ▸ *b*). Both the driving belt and the bearing of the rotation axis are embedded in the beam so that they do not make contact with cold nitrogen gas. Since there was no commercially available liquid-nitrogen Dewar with an appropriate size for SPACE-II, a special Dewar was manufactured (Soumei-Arts, Japan). The Dewar is wrapped in polyurethane foam, and it is further enclosed by the side panels of the storage chassis, to which rubber heaters are attached to prevent condensation. Liquid nitrogen is automatically supplied by a liquid-nitrogen generator (Taiyo Nippon Sanso), which is installed inside the experimental hutch. The apparatus generates liquid nitrogen by refrigerating nitrogen gas separated from the atmosphere. Since ice accumulates during the operation of SPACE-II, the Dewar is emptied and dried every two weeks. The positions where Uni-Pucks are installed are indicated by users using a graphical user interface (GUI) when they load Uni-Pucks. The GUI software is briefly explained in Section 2.5.

The top of the sample-storage chassis is covered with two lids (Fig. 1 ▸, Supplementary Fig. S1). To load a sample cassette into the Dewar, the lid at the distal side is removed manually. The other lid near the goniometer has a widow of 40 × 100 mm through which the twin arms access samples in the Dewar. This lid is further covered with a cover plate that is attached to the main part of SPACE-II and sits still during the translation of the Y-tray axis. The cover plate has a small lid that closes when the mount arms are oriented to the gonio­meter and opens when the mount arms are oriented to the sample storage (Fig. 1 ▸).

### 2.4. Ancillary equipment for SPACE-II   

A dryer is implemented for each arm to remove frost growing on the sample holder. A cylindrical nozzle for hot dry air is extended into the space between the goniometer and the sample storage (Fig. 1 ▸). The mount arms are inserted into the nozzles for drying. To completely remove water droplets after drying, an air blower is equipped under the dryer nozzle (not shown in Fig. 1 ▸). We recommend that users dry the mount arms every hour. This does not affect the throughput; because the sequence of the drying process is independent from the operation of the diffractometer, it can be conducted during sample centering or data collection.

To determine whether mounting/dismounting a sample pin has been successful, a CMOS laser sensor (LR-ZH500N; Keyence, Japan) was equipped on the goniometer (Fig. 2 ▸). In case of failure, a retry sequence proceeds automatically. The reasons for failures are described in Section 3.

Another important piece of equipment for SPACE-II is a safety light curtain (GL-R32H; Keyence, Japan), which is located in a space near the sample storage that users access to load/unload Uni-Pucks (Supplementary Fig. S2). It was implemented in order to check that a user does not come too close to SPACE-II, as the motion of SPACE-II is so rapid that it is dangerous for a user to come near. The output signal from the safety curtain is monitored at an interval of 30 ms so that it can immediately stop SPACE-II when the safety light curtain senses something move across it during any motion made by SPACE-II.

### 2.5. Control of SPACE-II   

SPACE-II is operated by server software (the *SPACE* server) running on the Microsoft Windows operating system. Each motor axis is controlled via a pulse-motor control card (PEX-H741280; Interface, Japan). The newly implemented direct-drive motor for the T-arm and robo-cylinder for the L-head are controlled through an IAI controller that accepts pulse signals generated from the PEX-H741280. Encoder pulses from the T-head and the spring sensor of the L-head are imported via a pulse-counter board (PCI-6204; Interface, Japan). The signals from the CMOS laser sensor used to detect sample pins on the goniometer are obtained via a DIO-0808LY-USB (Contec, Japan).

The software to control SPACE-II was inherited from the previous model. Briefly, a server program that controls SPACE-II was written using the C++ language. The sequential action of SPACE-II is defined by job-control scripts (JCSs), which are specialized text-based language scripts for the control of SPACE. A series of JCSs were prepared to mount/dismount samples and so on. The JCSs are interpreted and executed by the *SPACE* server when it received a corresponding command from a client. The use of JCSs has the advantage that we can modify the action without restarting the server software. In order to control SPACE-II, the *SPACE* server was modified to control newly implemented devices such as the direct-drive motor for the T-arm, the CMOS laser sensor and so on. New JCSs were also prepared to exchange a sample using the twin arms. For the user operation of loading and unloading Uni-Pucks, a graphical user interface was prepared using the Microsoft Visual C++ framework. It communicates with the *SPACE* server using a TCP/IP socket. The data-collection software *BSS* (Ueno *et al.*, 2005 ▸) also communicates with the *SPACE* server via TCP/IP socket communication to conduct sample exchange in users’ experiments.

### 2.6. Alignment and teaching   

Accurate alignment and proper teaching of SPACE-II are important for stable sample exchange. The outer diameter of the magnet base at the tip of the goniometer is 9.3 mm (https://hamptonresearch.com/uploads/support_materials/magnetic_drawing.pdf) and that of the magnet bases on a Uni-Puck base plate is 9.5 mm (http://smb.slac.stanford.edu/robosync/Universal_Puck/Universal_V1puck.pdf), whereas the inner dimeter of the base of the sample pin is 9.7 mm (see, for example, http://journals.iucr.org/d/issues/2006/10/00/gx5085/gx5085sup1.pdf). This means that a positional accuracy of better than ±0.1 mm is required for the mount arms of SPACE-II. This is achieved by the following alignment and teaching process in advance of user operation.

The first step is the alignment of the mount-arm direction towards the goniometer. Firstly, the horizontal orientation of the SPACE-II hardware around the normal to the diffracto­meter table is adjusted so that Arm-1 directing to the gonio­meter becomes parallel to the spindle axis. The T-arm angle is then adjusted so that the vertical orientation of Arm-1 becomes parallel to the spindle axis. For these alignments, a tool whose flat surface is confirmed to be parallel to the spindle axis is attached to the goniometer (Supplementary Fig. S3*a*), and the parallelism of Arm-1 to the spindle axis is verified by visual inspection. After this, a sample pin is attached at the tip of Arm-1, and it is extended into the position where the sample-pin base is about 1 mm distant from the magnet base on the goniometer. The translation stages of the goniometer *x* and *z* axes, which are perpendicular to the spindle axis, are adjusted so that the magnet base is placed at the center of the bottom concave of the sample pin held by Arm-1. These *x* and *z* positions of the goniometer are recorded as the reference positions to which *BSS* moves them prior to sample exchange. (The *y* position along the spindle axis is predetermined as the position in which the sample loop on the goniometer can be seen at the center of the on-axis microscope.) Arm-2 has two degrees of freedom relative to Arm-1: a tilt angle along the T-arm rotation direction and the positioning of Arm-2 along the L-switch axis after switching. Using these mechanisms, the vertical and horizontal positions of Arm-2 when it is extended to the goniometer are adjusted so that it locates just in front of the sample pin mounted on the goniometer.

The next step is the alignment for picking up/returning the sample pin from/to a Uni-Puck. The angle of the T-arm at which Arm-1 becomes normal to the rotation disk is determined using a square tool placed on the rotation disk (Supplementary Fig. S3*b*). Teaching of sample positions is performed by using a special tool designed for this purpose (Supplementary Fig. S3*c*). The tool has the same diameter as the Uni-Puck and has 16 holes corresponding to each sample-pin position of the Uni-Puck. Attaching this tool to the rotation disk, the Theta-tray and Y-tray positions where Arm-1 is located at the center of each hole are determined. This calibration is only necessary for a single Uni-Puck position, as the sample positions for the other seven Uni-Pucks can be extrapolated.

This alignment and teaching is performed just after the installation of SPACE-II on the beamline. After this, the sample-pin positions in the sample storage are checked every half year using the tool described above and corrected if necessary.

### 2.7. Sequence of sample exchange using twin arms   

**Figure 6:**
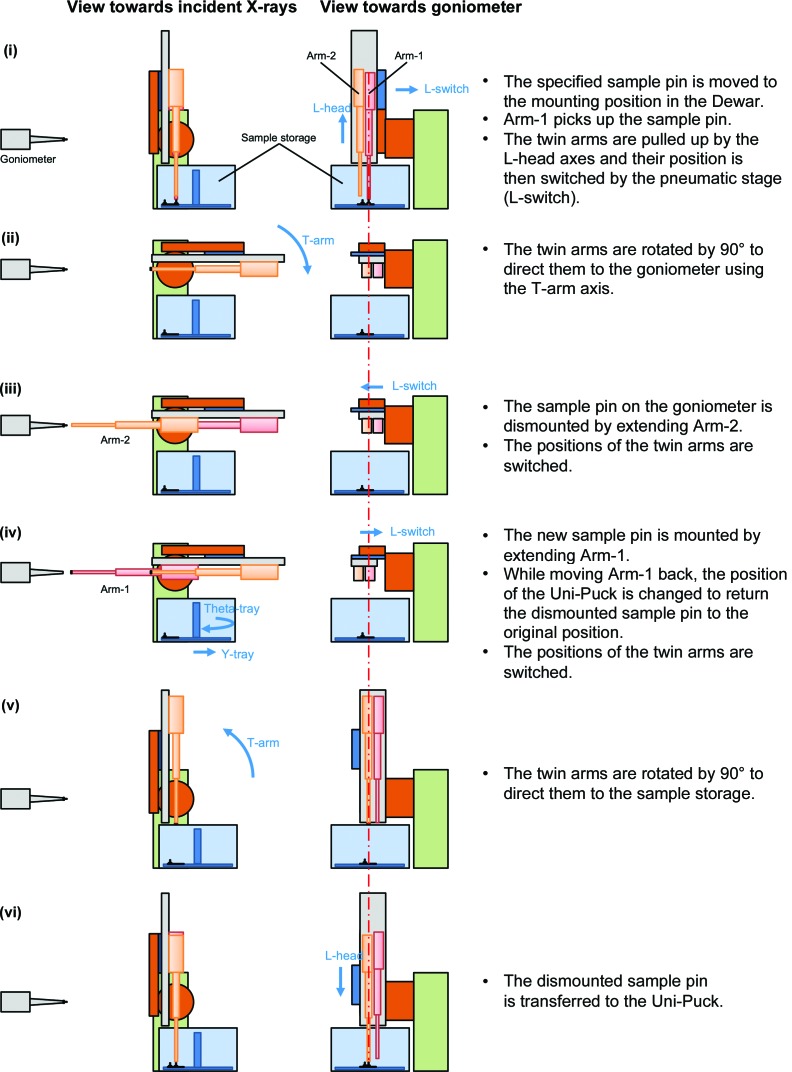
An illustration of the sample-exchange sequence performed by SPACE-II. The red dotted–dashed line indicates the position of the goniometer spindle axis to clarify the positions of the twin arms.

The sequence for exchanging samples using the twin arms is as follows and is also shown in Fig. 6 ▸ and Supplementary Movie S1. (i) Move the specified sample pin to the mounting position in the Dewar, (ii) pick up the sample pin with Arm-1, (iii) pull up the twin arms and change their position using the L-switch axis, (iv) orient the twin arms to the goniometer, (v) remove the sample on the goniometer by extending Arm-2, (vi) switch arms using the L-switch axis, (vii) mount the new sample by extending Arm-1, (viii) switch arms using the L-switch axis, (ix) orient the twin arms to the sample storage and (x) release the old sample into its original position on the Uni-Puck.

## 3. Performance of SPACE-II at BL41XU   

**Figure 7:**
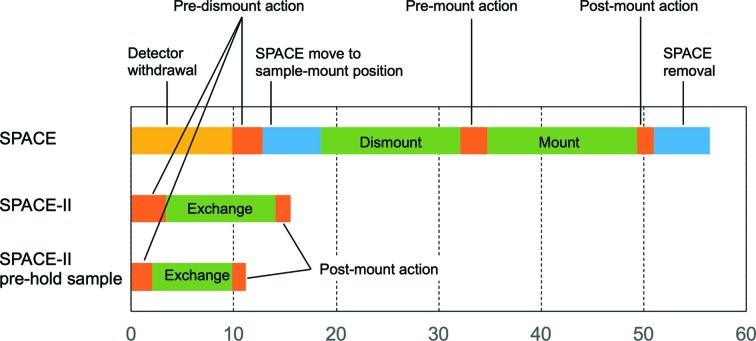
Comparison of the timeline for sample exchange between SPACE and SPACE-II. The timeline starts just after clicking the ‘OK’ button in the final confirmation window for sample exchange and finishes just before the time when users can start crystal centering. ‘SPACE-II pre-hold sample’ corresponds to the timeline in which Arm-1 holds the next sample prior to the start of the sample-exchange sequence. The time for detector withdrawal in SPACE corresponds to the time needed to change the camera length from 150 to 600 mm. Pre-dismount action includes the movement of the goniometer to the sample-mounting position and the removal of the cryo-stream. It also includes confirmation that devices such as the collimator and beam stop have been removed, which are automatically removed after each measurement. Pre-mount action in SPACE includes the confirmation of device removal, which could have been omitted by modification of the data-collection software *BSS*. Post-mount action includes insertion of the cryo-stream. The times were estimated from the log files of *BSS*.

SPACE-II was first installed at BL41XU and has been open for user operation since April 2017. The time taken to exchange samples was reduced from 56 to 16 s by the development of SPACE-II (Supplementary Movie S1, Fig. 7 ▸). The ‘time’ mentioned here is defined as the time from clicking the ‘OK’ button in a final confirmation window for sample exchange to the time when users can start crystal centering. Therefore, it includes pre- and post-mount actions, details of which are given in the caption to Fig. 7 ▸. The time taken for the action of SPACE-II itself is only 11 s of the 16 s.

A detailed timeline of sample exchange is shown in Fig. 7 ▸ together with that for SPACE. The sample-exchange time was reduced as follows. (i) There is no overhead owing to the withdrawal of the detector and the insertion/removal of the sample changer, which reduces the time by 21 s. This is accomplished by the implementation of the long-stroke L-head axis, which allows SPACE-II to be located far enough from the gonio­meter so that the detector never interferes with SPACE-II. In the case of SPACE, it takes 10 s for detector withdrawal and 11 s for the insertion/removal of SPACE. (ii) The speed of the L-head is increased from 200 to 465 mm s^−1^ by using the robo-cylinder. It sufficiently compensates for the 1.35-fold increase in the L-head stroke, so the time for the movement of the full stroke becomes almost halved. The speed of the T-arm is also increased from 40 to 200 deg s^−1^. (iii) The implementation of the twin arms allows samples to be exchanged in one cycle. The overhead owing to the switch of the two arms is less than 1 s, which is sufficiently small compared with the gain obtained from implementing the twin arms. The combination of (ii) and (iii) reduces the time by another 19 s.

To confirm whether the efficiency of beamline operation was improved after the installation of SPACE-II, the number of sample pins exchanged by SPACE-II between April 2018 and February 2019 (FY2018) and the number exchanged by SPACE between April 2016 and December 2016 (FY2016) were compared (Table 1 ▸). The average and maximum number per day were 132 and 290, respectively, in FY2016, and these numbers increased to 185 and 371 in FY2018, clearly demonstrating that SPACE-II contributed to efficient beamline use. The numbers of errors in these periods are also shown in Table 1 ▸; in FY2018, SPACE-II mounted 23 525 samples, of which 29 could not be mounted or dismounted owing to failure, meaning that the error ratio was 0.12%, which is slightly larger than the 0.078% error ratio in FY2016. We defined failures as incidents in which users could not continue an experiment without recovery work that involved the staff or the users themselves entering the experimental hutch. (i) Nine of the errors were failures to release the sample pin on the goniometer or Uni-Puck. In most of these cases, the arms were not dried for more than one hour, which is our recommendation, and ice at the tip of the mount arm might have caused the problem. (ii) Five of the errors were failures to pick up sample pins from Uni-Pucks. This is likely to be caused by ice that might be attached between the sample pins and Uni-Pucks when loading samples. (iii) Five of the errors were caused by improper loading in the Dewar, the use of broken Uni-Pucks, or defective sample pins. (iv) One error was caused by the improper positioning of the CMOS laser sensor for detecting samples on the goniometer, while another was owing to a bug introduced into the SPACE-II control software during speed optimization. Both of these errors have now been fixed and have never occurred again. There was also a failure caused by the formation of ice connecting the two arms because they were inserted into liquid nitrogen for more than eight hours without any operation. This also should not happen again because the arms are now forced to be dried if they have been continuously inserted in liquid nitrogen for more than 50 min. (v) The causes of the seven other errors were not clear. Since (iii) and (iv) are not caused by SPACE-II itself, the number of failures in FY2018 could be said to be 21, meaning that the error rate was 0.089%.

## 4. Discussion and conclusion   

The reduction of sample-exchange time is one of the most crucial steps in maximizing the throughput of MX beamlines, because diffraction data collection itself is completed within a few minutes in the era of pixel-array detectors. The development of SPACE-II has enabled samples to be exchanged in 16 s, where the action of SPACE-II accounts for 11 s. As far as the authors know, it is one of the fastest sample exchangers at MX beamlines worldwide. The performance of SPACE-II at BL41XU has clearly demonstrated that it contributes to efficient beamline use, as shown in Table 1 ▸. This rapid sample exchange was achieved with three major upgrades from SPACE: (i) the implementation of long-stroke mount arms that eliminate the withdrawal of the detector before sample exchange and removal of SPACE-II after sample exchange, (ii) the use of a fast-moving rotation and translation stage for the mount arms and (iii) the implementation of twin arms that enable samples to be exchanged in one cycle. The important point in this achievement is that unlike multi-axis robots, SPACE-II has only a limited degree of freedom in its motion, which allows the creation of a setup in which the mount arms never interfere with the detector. Therefore, sample pins can be exchanged without withdrawal of the detector by the quick rotation and translation of the mount arms.

The advantage of the twin arms is not only that sample exchange is completed in one cycle, but also that the arm can pick up a new sample before finishing data collection with the previous samples, which could further reduce the time needed for sample exchange. This functionality has already been implemented in our data-collection software *BSS* and tested at BL41XU, demonstrating that the time for sample exchange was further decreased to 11 s, in which the action of SPACE-II accounted for 8 s (Fig. 7 ▸). This is useful for automatic data collection in which the precise timing of sample exchange is predictable.

One of the most important things for sample changers at MX beamlines is reliability, because problems with the sample changer could waste limited beam time on recovery and, in more serious cases, invaluable samples might be spoiled. From this point, the error rate of 0.089% is quite low, so SPACE-II could be said to be quite reliable. This high reliability could be attributed to its simple architecture and the accumulation of small improvements to hardware and software based on the long experience with SPACE. On the basis of its success on BL41XU, SPACE-II was installed on BL44XU in August 2018 and on BL26B2 and BL45XU in April 2019.

Although SPACE-II achieves rapid and stable sample exchange, there is still one issue to be improved: the capacity of the sample storage. In SPACE-II this was increased to eight pucks, but this is not sufficient; at the highest exchange rate of 15.5 samples per hour in FY2018, it takes only 8 h to completely measure the samples stored in a Dewar. This would become a large problem for automatic data collection performed overnight when using the automatic data-collection system *ZOO* (Hirata *et al.*, 2019 ▸). However, a further increase in the sample storage is not realistic because doing so would enlarge the size of SPACE-II and it could not be located on the existing diffractometer table. Therefore, two other approaches to cope with this problem are being considered. One approach is to use sample pins that can be loaded more densely than with a Uni-Puck, such as the SPACE screw pin (Ueno *et al.*, 2004 ▸), miniSPINE and NewPin (Papp, Rossi *et al.*, 2017 ▸). However, none of these are widespread yet in the user community, at least in Japan. Therefore, an alternative approach is under consideration; that is, developing a puck exchanger together with a large puck-storage system.

In summary, we developed SPACE-II to enable rapid and reliable sample exchange. Operation over two years at BL41XU has demonstrated that SPACE-II achieves sample exchange in 16 s with an error rate of only 0.089%, contributing to the efficient use of beamtime. SPACE-II has now been installed on three other MX beamlines, BL26B2, BL44XU and BL45XU, at SPring-8. We conclude that SPACE-II is one of the most important pieces of infrastructure for MX beamlines at SPring-8, providing users with the opportunity to fully make use of the limited beamtime with brilliant X-rays.

## Supplementary Material

Supplementary Figures. DOI: 10.1107/S2059798320000030/wa5125sup1.pdf

Supplementary Movie S1. The sequence for exchanging samples by using the twin arms. DOI: 10.1107/S2059798320000030/wa5125sup2.mp4

## Figures and Tables

**Table 1:** Comparison of performance between SPACE and SPACE-II

	SPACE	SPACE-II
Period	FY2016 (April 2016 to December 2016)	FY2018 (April 2018 to February 2019)
No. of days†	127	130
Total No. of mounted samples	16708	23525
Average	132 per day (5.5 per hour)	185 per day (7.7 per hour)
Maximum	290 per day (12 per hour)	371 per day (15.5 per hour)
No. of failures‡	13 (6)	29 (21)
Percentage of failures‡	0.078 (0.035)	0.120 (0.089)

† Estimated by dividing the total number of experimental hours using SPACE or SPACE-II by 24.

‡ The values in parentheses indicate the failures caused by each robot. Precise definitions are given in Section 3.
