# Supplementary material for: Development of SPACE-II for rapid sample exchange at SPring-8 macromolecular crystallography beamlines
Source: Acta Crystallogr D Struct Biol. 2020 Jan 31;76(Pt 2):155–65. doi: 10.1107/S2059798320000030 (PMC7008514; doi:10.1107/S2059798320000030)
Supplement: Supplementary file 1 [file d-76-00155-sup1.pdf]

STRUCTURAL
BIOLOGY

Volume 76 (2020)

Supporting information for article:

Development of SPACE-II for rapid sample exchange on SPring-8 MX beamlines

Hironori Murakami, Kazuya Hasegawa, Go Ueno, Naoto Yagi, Masaki Yamamoto and Takashi Kumasaka

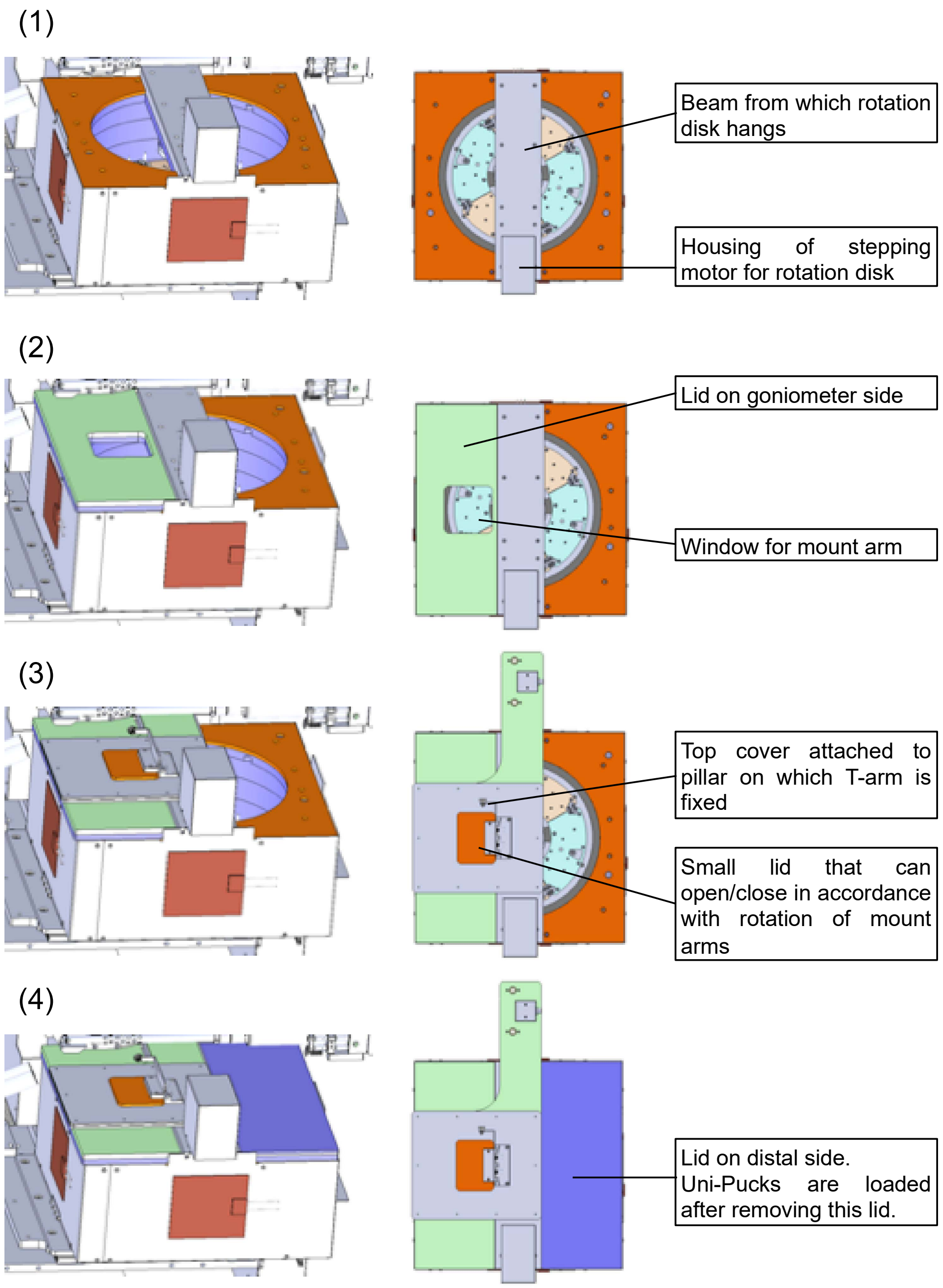

Figure S1 Assembly step of lids and cover plate of sample storage chassis.

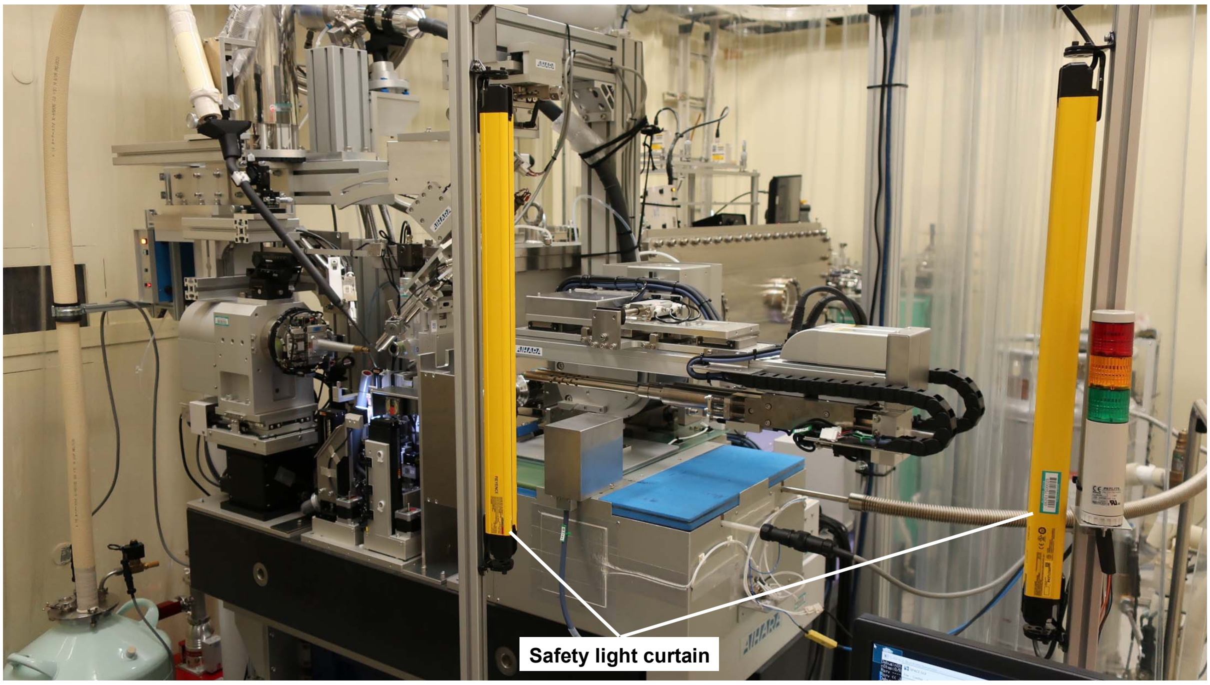

Figure S2 Implementation of safety light curtain to stop action of SPACE-II immediately when something crosses area between two yellow bars while SPACE-II is working.

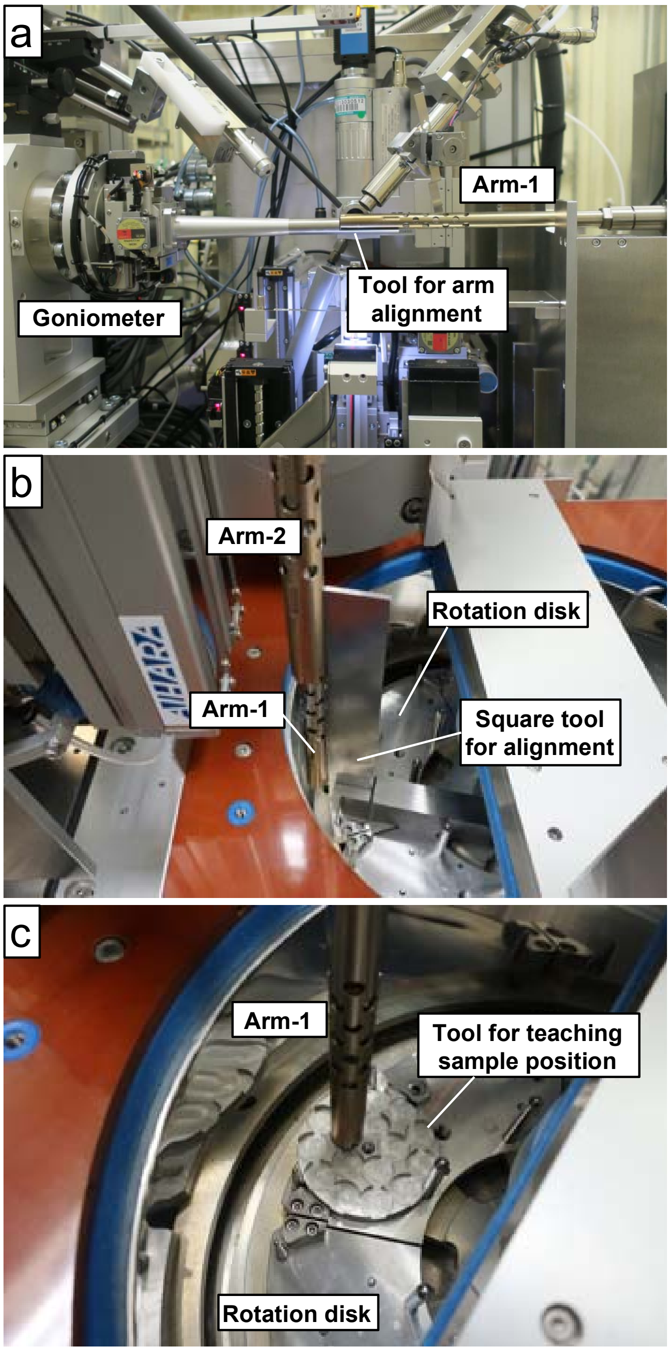

Figure S3 Tools for alignment and teaching of SPACE-II. (a) A tool to adjust orientation of Arm-1 direction to the goniometer. (b) A tool to adjust orientation of Arm-1 direction to the sample storage. (c) A tool for teaching sample position on a Uni-Puck. Teaching of sample positions is performed in the presence of liquid nitrogen whose level is just above the tool. The liquid nitrogen is removed in the figure for clarity.
